# Supplementary material for: Identification of photoperiod-induced specific miRNAs in the adrenal glands of Sunite sheep (Ovis aries)
Source: Front Vet Sci. 2022 Jul 22;9:888207. doi: 10.3389/fvets.2022.888207 (PMC9354845; doi:10.3389/fvets.2022.888207)
Supplement: Supplementary file 1 [file Data_Sheet_1.ZIP › Supplementary file/miRDeep2 file.zip!/miRDeep2 file/Know_Structure/SPLP42A2/oar-mir-23b.pdf]

miRBase precursor : oar-mir-23b
Total read count : 21890
oar-miR-23b read count : 21871
remaining reads : 19

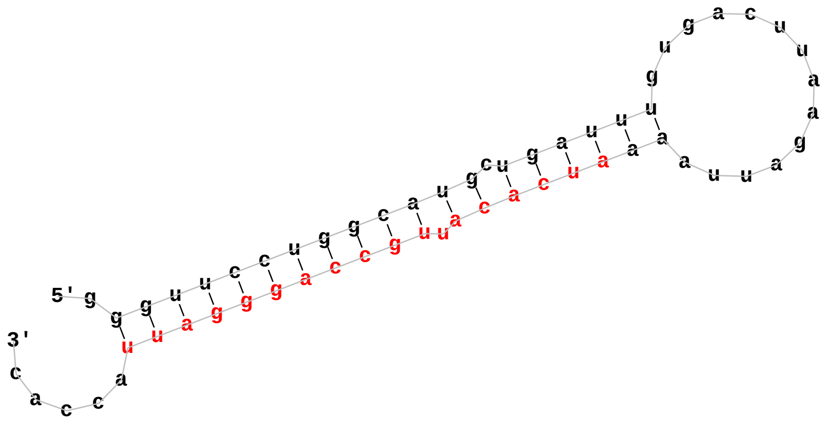

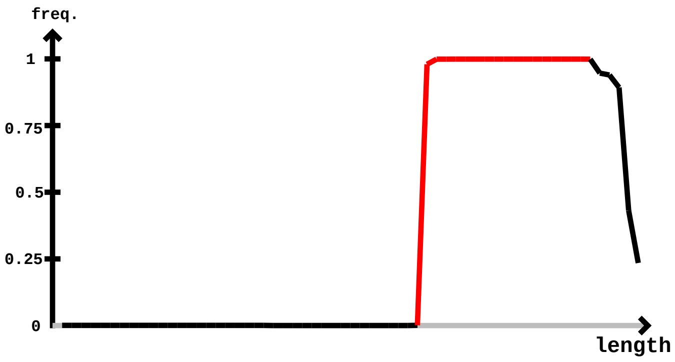


```
                                     oar-miR-23b
5'- ggguuccuggcaugcugauuugugacuuaagauuaaaaucacauugccagggauuaccac   -3'    exp
    ((((((((((((((.((((((..............)))))))).))))))))))..))..    reads      mm        sample
    ggguuccuggcaugcugauu........................................    3          0         seq
    ggguuccuggcaugcugauuu.......................................    13         0         seq
    ggguuccuggcaugcugauuugu.....................................    1          0         seq
    ....................ugugacuuaagauuaaaa......................    2          0         seq
    ....................................aaucacauugccagggauu.....    5          0         seq
    ....................................aaucacauugccagggauuac...    1          0         seq
    ....................................aaucacauugccagggauuacc..    10         0         seq
    ....................................aaucacauugccagggauuaccac    3          0         seq
    .....................................aucacauugccagggau......    8          0         seq
    .....................................aucacauugccUgggauu.....    1          1         seq
    .....................................aucacauugccagggCuu.....    3          1         seq
    .....................................aucacauugccagggauu.....    1121       0         seq
    .....................................aucacauugcUagggauu.....    3          1         seq
    .....................................aucacauugccagggauua....    150        0         seq
    .....................................aucacauugcUagggauua....    1          1         seq
    .....................................aucacauugccagggauuac...    999        0         seq
    .....................................aucacauugccagggauuacc..    9965       0         seq
    .....................................aucacauugccagggauuacca.    4218       0         seq
    .....................................aucacauugccagggauuaccac    4955       0         seq
    ......................................ucacauugccagggauu.....    10         0         seq
    ......................................ucacauugccagggauua....    1          0         seq
    ......................................ucacauugccagggauuac...    24         0         seq
    ......................................ucacauugccagggauuacc..    125        0         seq
    ......................................ucacauugccagggauuacca.    85         0         seq
    ......................................ucacauugccagggauuaccac    177        0         seq
    ........................................acauugccagggauuacc..    2          0         seq
    ........................................acauugccagggauuacca.    2          0         seq
    ........................................acauugccagggauuaccac    2          0         seq
```
